# Industrial Phthisis

**Published:** 1902-09-06

**Authors:** 


### Hospital Clinics and Medical Progress.

### INDUSTRIAL PHTHISIS.

In a discussion which took place at Manchester on the relation of phthisis to factory and workshop conditions, several interesting matters were brought out. Dr. McLeary, of Manchester, drew attention to a serious defect in our system of death registration in regard to women, namely, the common practice of registering the occupation, not of the deceased, but of her father or husband, a practice which enormously detracts from the value of the returns for public health purposes; and he instanced the difficulty of proving the frequency of phthisis among laundresses owing to this defective system. So much valuable information has been derived from the registration returns in regard to male occupational mortality, that it seems a great pity that we should be debarred by such a curiously stupid arrangement from obtaining similar information concerning the fatality accompanying the various occupations in which women are engaged.

Another point raised was in regard to gannister-miners' disease. In certain manufacturing processes, such, for example, as in the lining of Bessemer converters, a substance is required which will resist a very high temperature, and such a substance is gannister, a hard, close-grained mineral, consisting chiefly of silica, and it is the dust arising from the mining of this mineral which is responsible for gannister disease. There would seem to be a close resemblance between the etiology of this malady and that of the disease which occurs among the men engaged in the deep gold mines of the Transvaal, to which we recently referred in these columns. According to Dr. Robertshaw, who spoke on the subject, the cases, so far as tuberculosis was concerned, might be classed under three heads:—(1) Those in which, after the fibroid change had damaged the lungs, true tuberculous phthisis played the chief part; (2) those in which, although tubercle was found, it was only secondary to the fibroid disease; and (3) those in which there was no tubercle at all, the disease being entirely due to fibroid change brought about by the gannister dust, the majority of the cases being found in the last two classes. It is difficult to know what to do to prevent the conditions which tend to this disease. The gannister has to be blasted with dynamite, every discharge of which disturbs the dust far and wide. Dr. Robertshaw suggests that after the "shots" have been fired sufficient time for the greater part of the dust to settle should be allowed to elapse before the men re-enter the workings, and that a limit should be put to the length of time during which any individual should be allowed to work at the more dangerous branches of the trade. Perhaps greater care to obviate accumulation of dust in the mines, together with free watering in the neighbourhood when blasting is going on—methods found useful in coal mining for the prevention of explosion from the ignition of coal dust—might prove of some avail in preventing disease among the gannister miners. As for the breakers and grinders we may presume that the discovery of some "wet" process ought not to be beyond the wit of man.

Another curious mode of infection with tubercle, and possibly with other diseases, was described by Dr. T. Watts. It appears that in cotton weaving, the method at present in vogue amongst weavers for threading the shuttles consists in what is popularly known as "sucking up the weft." When the shuttle runs empty the weaver puts in a new "cop" or bobbin, and then draws the free end of the weft through the eye of the shuttle by suction. For this purpose the shuttle is placed between the weaver's lips and a forced inspiration is made by which the end of the weft is drawn through its eye. Unfortunately, whatever else then may happen to be in the eye of the shuttle is also drawn into the mouth, and often, no doubt, into the respiratory passages, and when it is remembered that an operative will usually have charge of three looms, and that on an average this operation is done once for each loom every five minutes, it is evident that a risk so frequently repeated is sure in the end to lead to mischief. This risk is not only from the possible transference of infection from some other user of the shuttle, although this may readily occur when a weaver temporarily takes charge of the loom of another weaver who is suffering from consumption, but from the fact that the eye of the shuttle becomes charged with dirt, which partakes, of course, of the average impurities contained in the "size," the cotton, and the general dust of the room. Dr. Watts recommends the employment of a small indiarubber pump, by which sucking the weft can be done quite as readily as by the mouth. He had found it difficult to get the older hands to use it, but children and young persons took to it readily.

